# Exercise-induced venous thrombosis of the upper extremity: A case report

**DOI:** 10.1177/2050313X241253731

**Published:** 2024-05-17

**Authors:** Kathleen Trinh, Antonio M. Pessegueiro

**Affiliations:** Department of Medicine, David Geffen School of Medicine at UCLA, Los Angeles, CA, USA

**Keywords:** Paget-Schroetter syndrome, thoracic outlet syndrome, deep vein thrombosis

## Abstract

Paget-Schroetter syndrome, the venous variant of thoracic outlet syndrome, is an uncommon presentation of deep vein thrombosis. In patients with Paget-Schroetter syndrome, the subclavian vein is compressed within the thoracic outlet as a result of repetitive and vigorous arm motions. Repeated endothelial injury leads to stasis in flow and eventual thrombus formation in the subclavian vein and its tributaries. This report highlights the case of an active and otherwise healthy 46-year-old patient who presented with swelling and pain of his right upper extremity after a run and was found to have multiple, effort-induced thrombi involving the right subclavian, axillary, brachial, and basilic veins. The unusual clinical picture of Paget-Schroetter syndrome and its presentation commonly in the demographic of young, healthy individuals make it a diagnosis likely overlooked and unfamiliar to many in the clinical setting.

## Introduction

The thoracic outlet is a narrow space in the lower neck bound by the clavicle anteriorly and first rib posteriorly wherein the neurovascular bundle consisting of the subclavian vein, subclavian artery, and brachial plexus travels through.^1^ Venous thoracic outlet syndrome refers specifically to the compression and thrombosis of the subclavian vein. The compression of the subclavian vein is a phenomenon that can occur in normal individuals during straining positional changes involving the head, neck, and arm, which further narrows the thoracic outlet space.^2^ Paget-Schroetter syndrome (PSS) manifests when this process is intensified, which often involves a combination of factors including an underlying anatomical anomaly of the thoracic outlet and often engaging in activities that repeatedly evoke such straining positions.^3^ The increased degree and frequency to which the subclavian vein becomes injured results in eventual venous thrombosis. Patients with PSS may initially be unaware of their condition due to ensuing collateral veins that can ameliorate venous congestion.^4^ It is not until venous return is substantially obstructed by further propagation and development of thrombus in the collateral veins that patients present with a painful, swollen, and cyanotic upper extremity.^4^ Here we report the case of an otherwise healthy 46-year-old male firefighter who was found to have extensive thrombi involving the right subclavian, axillary, brachial, and basilic veins induced from his routine runs. We hope this case report will increase awareness of PSS as an uncommon presentation of deep venous thrombosis and encourage prompt diagnosis of this syndrome based on history and physical examination alone.

## Case

**Figure 1.**
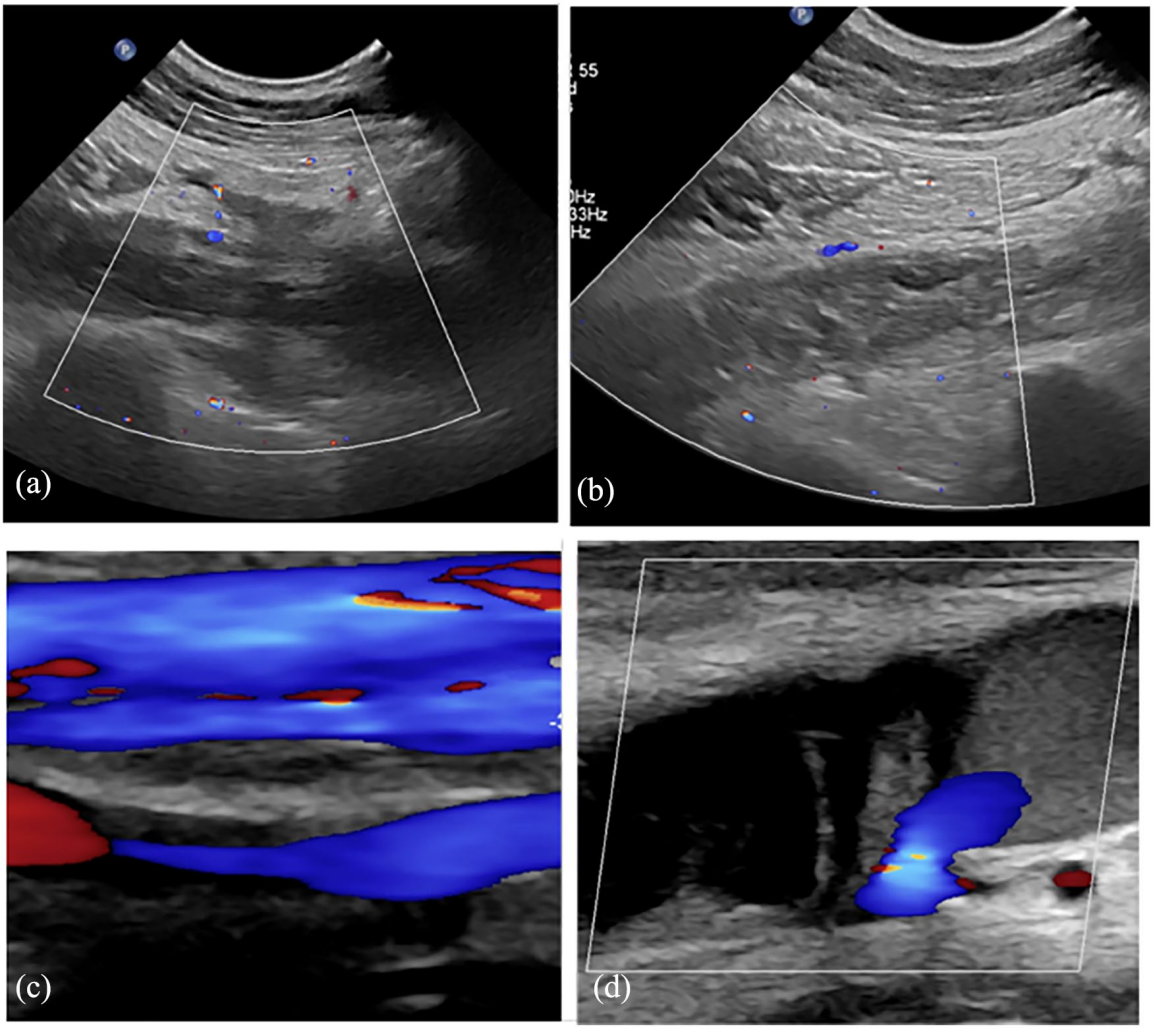
Initial assessment with duplex venous ultrasound of the right upper extremity demonstrating thrombi with resultant partial or no Doppler flow in the subclavian (a), axillary (b), paired brachial (top patent, bottom occluded) (c), and basilic (d) veins.

A 46-year-old male firefighter with no significant past medical history presented to the emergency department with increasing swelling and pain of his right upper extremity (RUE) for 1 day. The patient first noticed these symptoms while on his usual six-mile run the night prior and described worsening of both swelling and pain in his extremity upon running again the next morning. Initial duplex venous ultrasound of the RUE revealed extensive thrombus involving the right subclavian, axillary, brachial, and basilic veins (Figure 1). Computed tomography (CT) angiogram of the chest confirmed these findings and showed no evidence of pre-existing anatomical narrowing that would predispose him to thrombosis formation. The patient was evaluated for provoking risk factors such as prolonged immobility, recent trauma or surgery, obesity, malignancy, and known family history of thrombophilia—all of which were denied except for a strong family history of colon cancer (however he reported a normal colonoscopy performed at age 40). The patient’s lifestyle was overall extremely active as an avid runner and firefighter. He denied tobacco, alcohol, or drug use.

**Figure 2.**
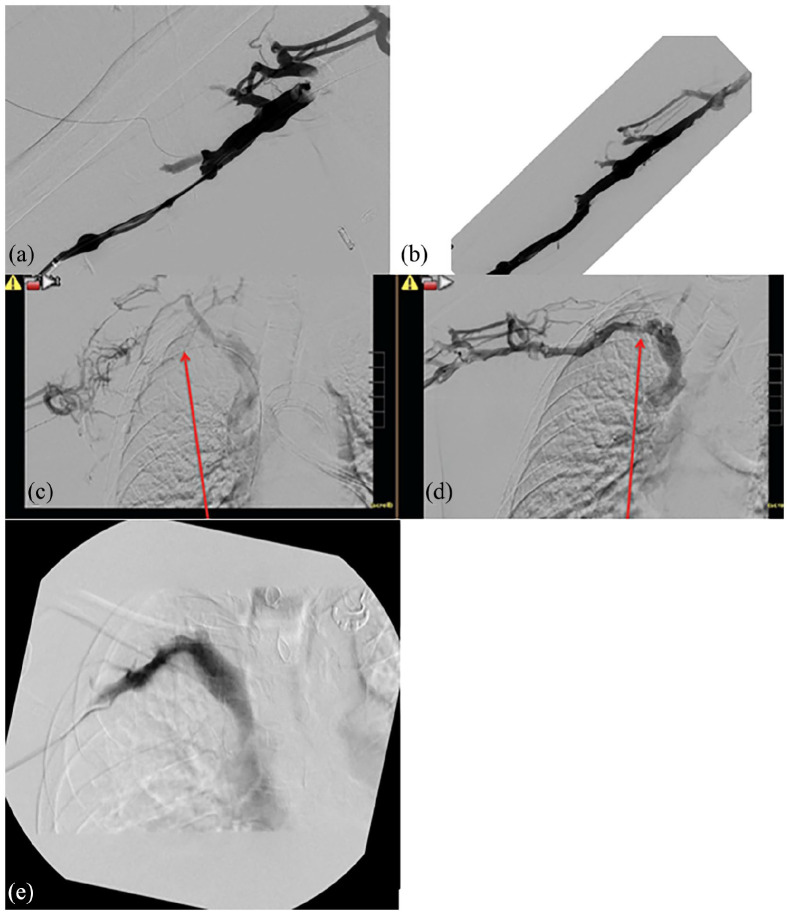
(a) Pre-lysis venogram showing decreased flow and engorgement of the upper extremity veins. (b) Post-lysis venogram demonstrating restored patency. (c) Pre-lysis venogram with arrow pointing to thrombus with collateral drainage. (d) Arrow shows outflow through the normal veins with decreased (but persistent) collaterals following lysis. (e) Venogram of right subclavian vein 2 months post-first rib resection surgery and repeat venoplasty showing patent flow without stenosis.

The patient was admitted to the hospital and initiated on therapeutic-dose anticoagulation with intravenous unfractionated heparin. He subsequently underwent catheter-directed thrombolysis within the RUE from the basilic to subclavian veins by interventional radiology to urgently restore patency of these vessels and mitigate his symptoms. A pre-lysis venogram was performed (Figure 2(a) and (c)). Thrombolysis was accomplished using a continuous infusion of tissue plasminogen activator at a rate of 0.5 mg/h through an infusion catheter positioned centrally in the right subclavian vein and peripherally in the basilic vein. He also remained on heparin infusion peripherally. He was monitored on therapy overnight on the medical-surgical unit per procedure and hospital policy at our institution. Approximately 20 h after initiating catheter-directed thrombolysis, he returned to the interventional radiology suite where post-lysis RUE venogram showed resolution of the previously seen thrombi (Figure 2(b) and (d)). It additionally demonstrated sluggish outflow and narrowing at the level of the first rib with the arm in abduction versus brisk outflow with the arm in adduction, consistent with venous thoracic outlet syndrome. A left upper extremity venogram was also performed and did not show evidence of venous thoracic outlet syndrome on the left. He lastly underwent gentle venoplasty of the RUE axillary and subclavian veins to address <50% residual stenosis in these vessels and remained on therapeutic-dose anticoagulation post-procedure. Subsequent consultation with vascular surgery yielded a recommendation for outpatient surgical decompression with resection of the right first rib to prevent future thrombosis. The patient was discharged on hospital day four on therapeutic-dose oral anticoagulation with apixaban.

The patient completed a hypercoagulable workup during an outpatient consultation visit with hematology, which included screening for factor V Leiden, prothrombin G20210A mutation, protein C deficiency, protein S deficiency, antithrombin III deficiency, and antiphospholipid syndrome. All studies resulted negative or within normal limits.

About 5 months after his hospitalization (due to patient availability and preference), the patient successfully underwent right first rib resection with vascular surgery with the goal to prevent future thrombosis. The right first rib resection was performed using a transaxillary approach and involved partial anterior middle scalenectomy. The patient remained on apixaban after surgery in anticipation of venoplasty for residual stenosis of the right axillosubclavian veins in the future. Two months after the initial surgery, the patient underwent repeat venogram and intravascular ultrasound of the RUE, which confirmed extensive post-phlebitic changes and residual stenosis in the right subclavian vein. He underwent repeat venoplasty of the right subclavian vein, resolving the stenosis (Figure 2(e)). Following the procedure, vascular surgery deemed it appropriate to discontinue apixaban.

## Discussion

PSS was initially termed and described as primary effort thrombosis by Sir James Paget in 1875 and later by Leopold von Schroetter in 1884.^3^ The phrasing of effort thrombosis implies a causal relationship between an exertion of bodily strain and subsequent thrombosis. As previously mentioned, subclavian venous anomalies can be present even in healthy subjects so as long as they were placed in aggravating positions, such as with the arm in abduction with a concurrently turned head.^2^ Such as our patient, those who are diagnosed with PSS are often younger, without significant health issues, and share a history of an active lifestyle, especially one with dynamic arm motion in their daily life. Of 34 reported patients with PSS in a 10-year span in Germany, eight patients demonstrated a relationship between formation of thrombosis to a sports activity. Repeated strain on the vein in different arm positions (e.g., hyperabduction, overhead maneuvers, etc.) lead to traumatic fissures in the intima and activation of coagulation, a process intensified by muscle contraction.^5^ A systematic review of PSS in athletes revealed an age range of 16–56 years with an average of 24 years, the majority of individuals were male (80.5%), and 32.5% had no past medical history.^6^

While thrombolysis (specifically if performed within 14 days of symptom onset^7^) and venoplasty may be successful in dissolving thrombi and restoring patency to the affected veins, definitive management is with thoracic outlet decompression surgery of the affected limb with first rib resection.^8^ Patients benefit from early surgical intervention as delays beyond weeks to months can result in fibrotic scarring in the affected vein, reducing its candidacy as a vessel that can be patched, dilated, or bypassed in the future.^4,9^ Those treated with surgical decompression additionally are more likely to be able to maintain their pre-existing functional and sporting capacity.^10^ Nonoperative treatment (e.g., with anticoagulation alone) as definitive therapy is generally not recommended, as it not only has an increased rate of failing to restore patency but is also especially prone to cause recurrent thrombosis.^10^ Appropriate management with complete thoracic outlet decompression therefore is strongly recommended to reduce the rate of re-thrombosis and long-term morbidity.

The presentation of spontaneous, multiple deep vein thrombi in an otherwise healthy and young patient without relevant medical history or risk factors would normally and appropriately warrant extensive workup in search for a provoking cause. This case highlights the importance of maintaining a high clinical suspicion for PSS, not only for healthy patients without identifiable risk factors but also highly active individuals who perform repetitive arm motions in their daily life (e.g., athletes, string instrumentalists, hairdressers, etc.). An increased awareness of the general demographic and presentation of PSS can lead to the reduction of delays in both diagnosis and treatment, allowing for more favorable outcomes in these patients.

## Conclusion

PSS occurs in the thoracic outlet due to repetitive compression of the subclavian vein, typically in healthy individuals without significant historical risk factors or medical history. Early recognition and definitive management with thrombolysis and decompression surgery lead to excellent outcomes. Increasing awareness of the presentation of PSS among all providers, including emergency room and primary team members, can enable prompt diagnosis, facilitating timely initiation of treatment.
